# Effect of L-Carnitine and/or Calf Thymus Gland Extract Supplementation on Immunity, Antioxidant, Duodenal Histomorphometric, Growth, and Economic Performance of Japanese Quail (*Coturnix coturnix japonica*)

**DOI:** 10.3390/vetsci8110251

**Published:** 2021-10-26

**Authors:** Ibrahim S. Abu-Alya, Yousef M. Alharbi, Hassan A. Abdel-Rahman, Ibrahim S. Zahran

**Affiliations:** 1 Department of Physiology, Faculty of Veterinary Medicine, University of Sadat City, Sadat City 32897, Egypt; 2 Department of Veterinary Medicine, College of Agriculture and Veterinary Medicine, Qassim University, Buraydah 51452, Saudi Arabia; yhrby@qu.edu.sa; 3 Department of Physiology, Faculty of Veterinary Medicine, Aswan University, Aswan 11731, Egypt; Ebrahimzahran@vet.aswu.edu.eg

**Keywords:** quail, L-carnitine, thymus extract, performance, immunity, antioxidant, duodenal histomorphometric, economic profiles

## Abstract

This study aimed to evaluate the supplementation (of drinking water) effects of L-Carnitine (LC) and/or calf thymus extract (TE) on immunity, antioxidant, duodenal histomorphometric, growth, and economic performance of Japanese quail. Six hundred, one day old unsexed quail were divided into four groups, the control group (G1) received plain drinking water, while G2, G3, and G4 were supplemented with LC and/or TE, respectively. The treated groups recorded a significant (*p* < 0.05) improvement in the final body weight, weight gain, feed conversion ratio, phagocytic activity %, phagocytic index, lymphocytes %, and plasma glutathione level compared to the G1. On the other hand, these supplementations significantly lower the mortality rate %, heterophil %, H/L ratio and plasma malondialdehyde level of the supplemented groups relative to G1. The supplemented groups recorded a non-significant increase in duodenal histomorphometric parameters (villous height, villous width, surface area, and crypt depth) relative to G1. Generally, LC and/or TE improve the values of economic profile (total returns, net profit, total return/total costs %, etc.) in treated groups compared to the control one. In conclusion, L-carnitine supplementation was more effective than TE or their combination with TE in the immunological, anti-oxidative, growth, and economic performance.

## 1. Introduction

The introduction of Japanese quail into intensive poultry rearing systems could be an alternative to meet the increasing demands for poultry meat and eggs [1]. Quail meat and eggs are prized for their high protein content, biological value, and low calorie content [2].

The poultry meat industry has been continually seeking strategies to improve the performance of commercial flocks and reduce carcass fat deposition [3]. Due to its beneficial effects on enhancing resistance to metabolic diseases, preventing some diseases, boosting the immune system, and improving poultry performance, LC is being considered as an alternative feed additive in poultry diets [2]. L–carnitine is a quaternary amine (4-Ntrimethylammonium-3-hydroxybutyric acid) that is required for mitochondrial oxidation of long-chain fatty acids by for tissues oxidation and ATP production [4]. Carnitine could improve the utilization of fat as energy, and it could improve feed conversion and carcass traits. Panahi et al. [5] found that LC supplementation improves growth performance and food efficiency significantly in the broiler. On the other hand, no positive benefits of LC were recorded on the performance of quail [6] and layer [7].

The thymus gland is a main lymphoid organ that provides a location for T cell generation and activation, and is regarded as an important immune system organ [8]. With age and after puberty, as the thymus begins to shrivel and involute, the production and growth of T-cells decreases. Thus, thymus extracts play a significant role in the formation, maturation, activation, migration, and stimulation of interleukin-2 (IL-2) production of T cells throughout the body [6,9]. Hormones that govern immune function are also released by the thymus [10]. The primary components of thymus extract are thymalin and thymosin. Thymalin is a polypeptide complex isolated from the thymus gland that has been approved for use as an immunomodulatory agent by the USSR Ministry of Health Order No. 1008 of 10.11.1982, Registration No. 8.1008.8 [11]. Thymosins (soluble hormone-like peptides) are produced by the thymus gland and can mediate immunological and non-immune physiological processes. They have recently received interest as treatments for inflammatory and autoimmune illnesses [12]. The thymus extract has been licensed for the treatment of certain disorders in a few animal species (dogs, rabbits, and pigs) [13]. Thymic peptides were participated in inflammatory processes regulation as cytokine antagonists and act as medication for immune dysfunction [14]. Thus, the current research will look at the use of thymus extract as an antibiotic alternative to boost immune responses.

The purpose of this study was to determine the effects of L-carnitine and/or calf thymus gland extract supplementation (in drinking water) on immunity, antioxidant activities, duodenal histomorphometric, growth, and economic performance in Japanese quail (*Coturnix coturnix japonica*).

## 2. Materials and Methods

### 2.1. Birds and Housing

A 600 unsexed one day old Japanese quail chicks were used in the current experiment. Birds were randomly assigned into four equal groups. Chicks of each group were divided into three replicates and each replicate was housed in separated pen (1 m^2^). For making a deep litter system, we used wood shavings as the litter material. The birds were reared under 23.0 h light: 1.0 h dark. The environmental temperature was 37 °C for the first 2 days and then decreased stepwise by 3 °C at 4 days intervals to reach 21 °C. Birds were allowed ad libitum access to feed and fresh water. A commercial balanced quail starter ration containing 24.8% crude protein and metabolizable energy of about 2900 Kcal/Kg was used for feeding of the young birds (first two weeks of age). While adult quail were fed a diet containing 20.2% crude protein and 2800 kcal/Kg metabolizable energy. All experimental diets were formulated to meet the nutrient requirements for Japanese quail recommended by the National Research Council [15,16].

All bird handling procedures as well as sample collection and disposal were performed according to the regulations of Institutional Animal Care and Use Committee (IACUC), Faculty of Veterinary Medicine, Sadat City University, Egypt (Ethical approval number: vusc-007/1-21).

### 2.2. Supplemented Additives

**L-Carnitine:** It was obtained as a pure white powder from MEPACO Company (Arab company for pharmaceutical & Medicinal plants, Cairo, Egypt.)

**Thymus extract:** It was obtained from Biomedia (Company for Biological and Veterinary Products, Cairo, Egypt), under a trade name CYTOIMMUNE^®^. It is a liquid preparation, with each bottle having 500 mL capacity of calf thymus gland extract. The main ingredients of this extract are natural thymic peptides, thymalin, and polynucleotides.

### 2.3. Experimental Design and Feeding Program

The Japanese quail chicks were divided randomly into a control group and 3 treated groups each containing 150 chicks. The length of the experiment was 5 weeks. Each group was divided into three replicates with 50 chicks in each. The quail were supplemented with basic diets and all the treatments (LC and/or TE) administrated via drinking water as follows:

Group 1 (G1) received plain drinking water (without any additive) and considered as the control group.
Group 2 (G2) received LC at a dose of 50 mg/kg/day [17].
Group 3 (G3) received TE at a dose of 0.01 mL/bird/day as recommended by the supplier.
Group 4 (G4) received both LC and TE.

### 2.4. Growth Performance Parameters

The growth performance of Japanese quail was assessed by recording body weight gain, feed intake, and feed conversion ratio. Individual live body weights of quail were recorded at the beginning of the experiment and on a weekly basis afterward. Feed intake for each group was recorded weekly. The feed conversion ratio was calculated weekly as the amount of feed consumed per unit of body weight gain:

Mortality rate %: It was calculated according to the following formula:

Mortality rate (%) = [(I − E)/I] × 100.
where I = Initial number of the birds at beginning of experiments;
E = Number of birds at experiment end.

### 2.5. Blood Sampling

At the end of the experiment, 10 quail from each group were randomly selected, subjected to 6 h feed withdrawal and slaughtered. Blood samples were collected on heparinized tube for further analysis. Immediately, blood films were prepared for determination of phagocytic activity, phagocytic index; differential leukocytic count and heterophil (H)/lymphocyte (L) ratio (H/L ratio) within two hours of collection. After blood centrifugation at 3000 rpm for 15 min, clear plasma samples were separated and divided into 2 Eppendorf tubes/bird and immediately kept frozen at −20 °C until biochemical analysis of plasma antioxidant enzymes.

#### 2.5.1. Phagocytic Activity % and Phagocytic Index of Heterophils

Phagocytosis of polymorph nuclear cells was performed using *Candida albicans* and calculated according to the following formula [18]:

Phagocytic Activity % = (Number of heterophils ingesting candida/Total number of heterophils) × 100.
Phagocytic index = The total number of ingested candida/Number of active heterophils.

#### 2.5.2. Differential Leukocytic Count

Differential leukocytic count was calculated on Giemsa-stained blood smears [19].

#### 2.5.3. Plasma Antioxidant Enzymes

Plasma malondialdehyde levels were determined spectrophotometrically using commercial kits (Bio-Diagnostics Kits, CAT. No. MD 2529, Giza, Egypt) [20]. In addition, plasma reduced glutathione was determined spectrophotometrically using commercial kits (Bio-Diagnostics Kits, CAT. No. GR 2511, Egypt) [21].

### 2.6. Duodenum Micro-Morphological Measurements

The abdominal cavity was opened after slaughter, and the entire small intestine system was removed for histomorphological analysis. Two centimeters of mid-duodenum (from the gizzard exit to the end of the pancreatic loop) were removed, rinsed by physiological saline solution and preserved in 10% buffered formalin [22]. The tissue samples were later embedded in paraffin, and a 5 µm section of each sample was placed on a glass slide and stained with hematoxylin and eosin according to Baurhoo et al. [23] The tissue sections were examined by a Nikon phase contrast microscope coupled with a micro-computer integrated digital imaging analysis system (Nikon Eclipse 80i, Nikon Co., Tokyo, Japan). The height and width of 30 villi and the depth of 30 crypts were measured from each group. Villus height was measured from tip (with a lamina propria) of the villus to the base (villus-crypt junction) and villus width was measured at its middle part, while the crypt depth was measured from the villus-crypt junction to the distal limit of the crypt [24]. Finally, the values of micro-morphological measurements of 10 quail/group were expressed as mean ± SE. The surface area was calculated according to this formula [25]:

Surface area = (2π) × (VW/2) × (VL)
where VW = Villus width, VL = Villus length, π = 3.143

### 2.7. Economic Efficiency

#### 2.7.1. Costs of Production Were Classified into

Total Variable Costs,
Total Fixed Costs, and
Total Costs

Total Variable Costs (TVC) include feed costs, additives costs labor, litter, total veterinary management (drugs, vaccine, and veterinary supervision), water, energy costs (electricity and gas), and chick’s price. It was calculated with an Egyptian pound during the period of the experiment [26].

Total Fixed Costs (TFC) include building and equipment’s depreciation. Thus, these parameters were considered as a fixed value for all the experimental groups [26]. The depreciation rates were calculated for the building to serve for 25 years and for the equipment to be used for 5 years. The straight-line method implied by Kumar and Indira [27] was used for calculation of depreciation rates according the following equation:

Equipment depreciation = [Value of equipment (L.E)/Number of years]/Number of project cycles per year

Total Costs (TC) = Total variable costs + Total fixed costs

#### 2.7.2. Return Parameters

Total Returns (TR) were calculated according to following equation [28]:

Total Returns (TR) = Litter sale + Total quail sale;

Net Profit (NP): It was calculated using the following equation [26]:

Net Profit (NP) = Total Returns (TR) − Total Costs (TC).

#### 2.7.3. Economic Efficiency Measurements

- Percentage of total returns to total costs = (Total return/Total costs × 100);
- Percentage of net profit to total costs = (Net profit/Total costs × 100);
- Capital cycle = Investment costs/Net profit;
- Capita return rate = (Net profit/Investment costs) × 100.

### 2.8. Statistical Analysis

Comparisons of the mean of differences of LC and/or TE were analyzed using a one-way analysis of variance and Duncan’s post hoc test [29]. All statistical analyses were performed using the SPSS 23.0 software (SPSS Inc., Chicago, IL, USA). A *p*-value < 0.05 was considered statistically significant.

## 3. Results

### 3.1. Growth Performance and Mortality Rate %

As shown in Table 1, the supplementation of quail with LC and/or TE significantly (*p* < 0.05) improved the values of final body weight, final weight gain, and feed conversion ratio compared to the values of non-treated group (G1). On the other hand, feed intake among different groups did not differ from each other (*p* < 0.05).

### 3.2. Phagocytic Activity % and Phagocytic Index

Phagocytic activity % and phagocytic index were recorded a significant difference (*p* < 0.05) between the means of groups supplemented with LC and/or TE and the mean of the control group (Table 2). The best results of supplemented groups were obtained in G2 and G3 relative to the G4 value (combination of LC and TE).

### 3.3. Differential Leukocytic Count

As shown in Table 3, the LC and TE supplementation of G2, G3, and G4 had a significant decrease (*p* < 0.05) in heterophils %, H/L ratio, compared to the non-supplemented group (G1), while a significant (*p* < 0.05) higher lymphocyte % were recorded in groups supplemented with LC and/or TE relative to control one.

### 3.4. Antioxidant Profile

Plasma reduced glutathione contents were significantly increased (*p* < 0.05) in the LC and TE supplemented quail compared with those in non-supplemented ones. In contrast, the malondialdehyde levels were significantly (*p* < 0.05) depleted in all treated groups (G2, G3, G4) in relation to non-treated one (Table 4).

### 3.5. Duodenal Histomorphometric Parameters

As shown in Table 5, LC and/or TE supplementation (G2, G3, G4) in quail recorded a non-significant (*p* > 0.05) increase in duodenal histomorphometric parameters (villous height, villous width, surface area, and crypt depth) when compared to control group (G1).

### 3.6. Economic Parameter Traits

The calculated results in Table 6 indicated that the supplementation of quail with LC and/or TE of G2, G3, and G4 improve the values of total returns, net profit (return), total return/total costs percentage, net profit/total costs percentage, and capita return rate in treated groups compared to the control one (G1). On the other hand, these supplementations reduce the number of years needed by investors to uptake the invested capital in the farm (capital cycle).

## 4. Discussion

### 4.1. Growth Performance

The current findings showed that the LC and/or TE supplements improved final body weight and final weight gain. These results support Awad et al. [30] and Mahmoud et al. [31], findings that increasing levels of LC in growing Japanese quail improved bird body weight. The improvements in quail performance fed LC supplemented feeds may be attributed to improvements in the efficiency with which dietary long chain fatty acids are oxidized by mitochondria, and consequently to the energy released in such process [32]. Additionally, LC has been shown to increase plasma insulin-like growth factor I (IGF-I) concentration, which stimulates chick growth [33]. Beyond playing an anabolic effect on skeletal muscle, through increasing body protein accretion, IGF-I also stimulates the proliferation and differentiation of muscle stem cells (satellite cells). Previous evidence suggests that this process is essential for muscle hypertrophy [34]. Our outcomes, however, differ from those reported by Sarica et al. [35], who found no effects of dietary LC supplementation in Japanese quail body weight from 0 and 35 d of age. Likewise, Soliman et al. [10] did not notice the effects of TE associated or with LC in drinking water on broiler performance.

Our results showed that FCR was improved in quail supplemented with LC and/or TE and such outcomes were similar to those reported by Mahmoud et al. [31], who observed that growing quail fed diets supplemented with 200, 400, and 600 mg 279 LC/kg had improvements in FCR compared with those fed the unsupplemented control diet. This improvement may be related to the enhancement of LC to fatty acids burning, which lower calorie requirements, as well as improve intestinal mucous membrane by active and passive mechanism [36]. Additionally, birds supplemented with dietary LC showed a decrease in feed intake, and this feed reduction may be due to the ability of birds to compensate their feed reduction according to their energy requirements with similar metabolizable energy diets [30]. FCR improvement was previously linked to a reduction in feed intake and an increase in body weight gain in broiler chicks [37]. Our outcomes differ from Soliman et al. [10] and Xu et al. [38] findings who found that the administration of LC and/or TE in feeds and/or water did not affect broiler FCR.

### 4.2. Mortality Rate %

The present study concluded that water supplementation of LC and/or TE significantly (*p* < 0.05) reduce the mortality rate % in treated group in comparison with the control one. These data were approved by Daskiran and Teeter [39], who discovered that supplementing LC at levels of 40, 80, 120, 160, and 200 mg/kg diet increased livability by increasing more efficient fatty acid consumption in heart muscle. Inconsistency, Yalçin et al. [40] described that the dietary treatment of one week old Japanese quail with LC for four weeks did not reduce the mortality of quail whatever the dosage used. Similarly, some researchers found that mortality % of broilers [41,42] or quail [6] was not affected by LC supplementation.

### 4.3. Antioxidant Profile

L-carnitine has antioxidant properties by decreasing lipids availability for peroxidation via transportation of fatty acids into the mitochondria for β-oxidation to generate ATP energy [43,44]. The current study revealed that the water supplementation of LC and/or TE significantly (*p* < 0.05) increases the glutathione peroxidase level and reduces the malondialdehyde level in treated group compared to the control one. This finding was in accordance with those of Mahmoud et al. [31], who found that the diets supplemented with LC decrease the malondialdehyde level and increase catalase activity in hepatic tissue, breast, and thigh muscles, especially with the higher dose (600 mg/kg) in growing Japanese quail. Tian et al. [45] concluded that the LC has an exogenous anti-oxidant property by preventing the formation of reactive oxygen species, scavenging free radicals, and protecting cells from peroxidative stress. The present results were similar to that demonstrated by Mohammadi et al. [46] and Wang et al. [47]. Furthermore, LC, through its antioxidant properties, has been shown to increase the activity and levels of antioxidant enzymes such as superoxide dismutase and glutathione peroxidase in the poultry plasma [41].

### 4.4. Phagocytic Activity % and Phagocytic Index

The results of the present study revealed that the water supplementation with LC and/or TE expressively (*p* < 0.05) increase the phagocytic activity % and phagocytic index in treated groups compared to the control one. These results are in agreement with El-Gendy et al. [48] for male mice and Soliman et al. [10] for the broiler. Intraperitoneal injections of LC and/or TE increase phagocytic activity percent and phagocytic index in male mice [48]. However, the positive effect of calf thymus extract on humoral and cellular immunity may be attributed to increasing lymphocyte cells % [49]. The effect of LC can be linked to its potential to induce immunomodulatory effects, since supplementation boosts protective immunity after vaccination by increasing neutrophil and macrophage activity [45]. Cakir and Yalcin [50] found a putative positive effect of LC on humoral immunity of broilers receiving low energy diets. Janssen et al. [51] and Deng et al. [52] demonstrated that the LC supplementation enhance subsequent antibody responses in broiler pigeons and chickens. The LC immunomodulatory mechanisms may be contributed to white cell activation by enhancing their energetic metabolism through lipid oxidation [53] or by inducing secretion and release of immunomodulatory hormones such as insulin and insulin-like growth factor-I [54] and triiodothyronine [55]. Furthermore, LC may promotes lymphocyte survival by inhibiting apoptosis and by amplifying proliferative response to mitogens [56].

The present study agrees with the previous results which concluded that thymic peptide mixtures (Thymosin fraction 5, thymulin) have been proved to stimulate the immune response and enhance phagocytosis [57]. The increase in phagocytic activity of phagocytes in TE treated group could be due to its ability to act as an immunomodulator by exerting control on cytokine production by peripheral blood mononuclear cells [49].

### 4.5. Differential Leukocytic Count

The present study proved that the water supplementation by LC and/or TE markedly (*p* < 0.05) increases the lymphocyte % and decreases heterophils % and H/L ratio in the treated groups compared to the control one. These results are similar to the results of many authors [10,49,58]. Naik et al. [49] found that there was a marked significant increase in the number of lymphocytes in TE treated groups, when compared to the control one, which is one of the probable reasons for overall immunopotentiation, and this could be due to the fact that thymic hormones activate T-cell and activate lymphocyte production. Karadeniz et al. [58] published that the dietary LC supplementation had significantly increased the WBC_s_, heterophils, and lymphocytes counts. Awad et al. [30] declared that lymphocyte cells % were significantly increased for duckling fed diets supplemented with LC compared to the control one. In addition, the non-oral administration of LC decreases the N/L ratio compared to the oral route treated group [59]. Furthermore, Soliman et al. [10] reported that the supplementation of LC and TE increases lymphocyte % and decreases the H/L ratio due to decreased heterophil values in broilers. There was a considerably increased level of lymphocyte production in TE treated groups, and this could be due to the fact that thymic hormones activate T-cell rosettes, and they enhance the differentiation, maturation, and proliferation of lymphocytes [10]. It has also been reported that the injection of TE into thymectomized mice protects the fetus from viral infection, prevents the appearance of a wasting syndrome, and increases the number of lymphocytes in peripheral blood [60]. These results may be due to the existence of a diffusible product from thymus tissue in different strains and species, which enables thymectomized animals to produce new lymphocytes and to develop immunologic competence [61].

### 4.6. Duodenal Histomorphometric Parameters

Groups supplemented with LC and/or TE of the current study noted a non-significant (*p* < 0.05) increase on duodenal villus (length, width, surface area) and crypt depth relative to control group. Correspondingly, Zadeh Adamnezhad and GhiasiGhalehkandi [62] were not able to investigate any positive effects of selenium and/or vitamin E supplements on small intestine morphometry in the quail. On the other hand, micronized wheat fiber can be utilized as a feed additive in quail diets to increase performance, intestinal bacterial counts, and small intestine shape [63]. In addition, Kaolin inclusion in the feed resulted in a positive effect in quail performance and intestinal morphology [64]. These changeable results may be due to variable supplemented dosage, which was not sufficient to improve duodenal histomorphological parameters. In the same time, the observed differences in results may be related to ration composition, level, and type of supplementation, animal health status, age, and even strain [65,66]. The current results reported a non-significant (*p* < 0.05) increase of duodenal morphometry, but it was adequate for a significant (*p* < 0.05) increase of growth performance parameters (food intake, BWG, FCR, etc.) through enhancing food digestion and absorption in Japanese quail.

### 4.7. Economic Parameter Traits

Calculations of economic parameters traits listed in Table 6 revealed that the supplementation of quail with LC and/or TE in G2, G3, and G4 improve the values of total returns, net profit (return), total return/total costs %, net profit/total costs %, and capital return rate in treated groups relative to the control group. On the other hand, these supplementations reduce the number of years needed by investors to uptake the invested capital of the farm (capital cycle). In the same context, Awad et al. [67] described that the diet supplementation with different LC levels in Sudani ducklings increase the variable costs, total costs, total return, and net return values compared to the control group. In contrast, Warmazyar [68] concluded that the dietary supplementation with different levels of LC in broiler chickens has no significant effect on economic efficiency.

## 5. Conclusions

The supplementation of quail with LC and/or TE improved the growth performance, immunological, antioxidant, and economic performance traits. On the contrary, these supplements did not improve the duodenal histomorphometric parameters. The findings of the present research concluded that LC supplementation was more effective than TE or their combination with TE on the growth performance and economic performance of quail. Thus, these data can be used as basic information for the LC and/or TE to formulate a feed additive for Japanese quail rearing. Further research is needed to investigate effective levels of these supplements to correlate gut histomorphometric parameters and growth performance for the best economic profits.

## Figures and Tables

**Table 1:** Effect L-carnitine and/or calf thymus extract supplementation on growth performance parameters and mortality rate % in Japanese quail (mean ± SE).

	G1 (Control)	G2 (LC)	G3 (TE)	G4 (LC + TE)
Initial body weight (g)	7.12 ± 0.39 ^a^	7.09 ± 0.61 ^a^	7.25 ± 0.23 ^a^	7.13 ± 0.26 ^a^
Final body weight (g)	215.06 ± 1.90 ^c^	238.85 ± 2.52 ^a^	235.88 ± 2.35 ^a^	224.52 ± 2.43 ^b^
Final Weight gain (g)	207.94 ± 1.49 ^c^	231.76 ± 1.84 ^a^	228.63 ± 2.08 ^a^	217.39 ± 2.11 ^b^
* FCR	2.68 ± 0.024 ^a^	2.41 ± 0.016 ^c^	2.42 ± 0.018 ^c^	2.55 ± 0.020 ^b^
Feed intake (g)	565 ± 19.34 ^a^	559 ± 23.79 ^a^	554 ± 29.73 ^a^	555 ± 21.48 ^a^
Mortality rate %	9.22 ± 1.04 ^a^	4.44 ± 0.28 ^b^	5.00 ± 0.32 ^b^	5.33 ± 0.32 ^b^

^a–c^ Values with the different letter in the same row differ from each other at *p* < 0.05. * FCR = Feed Conversion ratio.

**Table 2:** Effect L-carnitine and/or calf thymus extract supplementation on phagocytic activity and phagocytic index in Japanese quail (mean ± SE), *n* = 10.

	G1 (Control)	G2 (LC)	G3 (TE)	G4 (LC + TE)
Phagocytic activity %	45.69 ± 0.52 ^c^	68.69 ± 0.46 ^a^	69.33 ± 0.73 ^a^	58.77 ± 0.67 ^b^
Phagocytic index	2.03 ± 0.050 ^c^	2.97 ± 0.026 ^b^	2.83 ± 0.040 ^a^	2.39 ± 0.031 ^b^

^a–c^ Values with the different letter in the same row differ from each other at *p* < 0.05.

**Table 3:** Effect L-Carnitine and/or calf thymus extract supplementation on differential leukocytic count % in Japanese quail (mean ± SE), *n* = 10.

	G1 (Control)	G2 (LC)	G3 (TE)	G4 (LC + TE)
Lymphocytes %	59.26 ± 1.25 ^b^	65.09 ± 1.38 ^a^	64.95 ± 1.52 ^a^	61.84 ± 1.25 ^ab^
Heterophils %	32.90 ± 0.95 ^a^	27.40 ± 0.58 ^b^	27.53 ± 0.42 ^b^	30.48 ± 0.81 ^ab^
Monocytes %	3.49 ± 0.36 ^a^	3.28 ± 0.15 ^a^	3.19 ± 0.22 ^a^	3.21 ± 0.20 ^a^
Eosinophils %	3.20 ± 0.31 ^a^	3.13 ± 0.16 ^a^	3.17 ± 0.39 ^a^	3.14 ± 0.42 ^a^
Basophils %	1.24 ± 0.12 ^a^	1.10 ± 0.14 ^a^	1.16 ± 0.05 ^a^	1.33 ± 0.06 ^a^
H/L ratio	0.56 ± 0.005 ^a^	0.42 ± 0.001 ^c^	0.42 ± 0.009 ^c^	0.49 ± 0.005 ^b^

^a–c^ Values with the different letter in the same row differ from each other at *p* < 0.05.

**Table 4:** Effect L-Carnitine and/or calf thymus extract supplementation on some plasma antioxidant profile in Japanese quail (mean ± SE), *n* = 10.

	G1 (Control)	G2 (LC)	G3 (TE)	G4 (LC + TE)
Malondialdehyde (mmol mL^−1^)	10.17 ± 0.09 ^a^	8.24 ± 0.49 ^b^	8.32 ± 0.22 ^b^	8.66 ± 0.33 ^b^
Reduced glutathione (mg dL^−1^)	29.61 ± 0.54 ^c^	35.42 ± 0.24 ^a^	34.99 ± 0.16 ^a^	32.31 ± 0.28 ^b^

^a–c^ Values with the different letter in the same row differ from each other at *p* < 0.05.

**Table 5:** Effect L-Carnitine and/or calf thymus extract supplementation on duodenal histomorphological parameters in Japanese quail (mean ± SE), *n* = 30.

	G1 (Control)	G2 (LC)	G3 (TE)	G4 (LC + TE)
Villous height (µm)	814.45 ± 61.81 ^a^	952.80 ± 67.02 ^a^	891.12 ± 59.78 ^a^	898.14 ± 60.46 ^a^
Villous width (µm)	110.36 ± 3.81 ^a^	112.86 ± 3.89 ^a^	111.53 ± 3.82 ^a^	111.56 ± 3.81 ^a^
Surface area (mm^2^)	277.71 ± 22.60 ^a^	347.01 ± 29.86 ^a^	321.38 ± 26.85 ^a^	299.29 ± 17.11 ^a^
Crypt depth (µm)	86.28 ± 2.15 ^a^	92.28 ± 2.10 ^a^	89.28 ± 2.35 ^a^	90.78 ± 2.12 ^a^

^a^ Values with the different letter in the same row differ from each other at *p* < 0.05.

**Table 6:** Effect L-Carnitine and/or calf thymus extract supplementation on * economic parameters traits ($) in Japanese quail (September 2021).

	G1 (control)	G2 (LC)	G3 (TE)	G4 (LC + TE)
Costs of water additives from day 1st to 35th day	-	7.62	12.71	20.33
Feed costs	296.57	293.02	290.41	290.92
Vaccines & drugs costs	12.71	12.71	12.71	12.71
Water & electrolyte costs	6.35	6.35	6.35	6.35
Labor costs	31.77	31.77	31.77	31.77
Other costs	127.07	127.07	127.07	127.07
Total variable costs	474.08	478.53	481.00	489.14
Building depreciation costs **^1^**	4.77	4.77	4.77	4.77
Equipment depreciation costs **^2^**	1.59	1.59	1.59	1.59
Total fixed costs	6.35	6.35	6.35	6.35
Total costs	480.43	484.88	487.36	495.49
Total weight sale **^3^**	576.87	607.37	603.56	601.02
Litter sale	6.35	6.35	6.35	6.35
Total returns	583.23	613.72	609.91	607.37
Net profit	102.80	128.84	122.55	111.88
Total return/total costs (%)	121.40	126.57	125.15	122.58
Net profit/total costs (%)	21.40	26.57	25.15	22.58
Capital cycle (years) **^4^**	1.85	1.48	1.56	1.72
Capita return rate (Cents) **^5^**	54.10	67.58	64.16	58.22

* Calculated on basis of each group contain 1000 quail chicks (Total No. = 4000) and dollar ($) value = 15.74 LE; **^1^** Calculated on the basis of building cost = 50,000 LE and depreciated on 25 years and the value divided into 4 groups and 7 production cycles/year; **^2^** Calculated on basis of equipment cost = 3500 LE and depreciated over 5 years and the value divided into 4 groups and 7 production cycles/year; **^3^** Average marketing price of one quail (Sept. 2021) = 10 LE (0.64$); **^4^** Determine the number of years that the investor needs to uptake the invested capital in the farm; **^5^** Determine the return of each dollar ($) invested in the farm.

## Data Availability

The data presented in this study are available on request from the corresponding author.
